# Overall mental distress and health-related quality of life after solid-organ transplantation: results from a retrospective follow-up study

**DOI:** 10.1186/1477-7525-11-15

**Published:** 2013-02-08

**Authors:** Andreas Baranyi, Till Krauseneck, Hans-Bernd Rothenhäusler

**Affiliations:** 1 Department of Psychiatry, University of Medicine of Graz, Auenbruggerplatz 31, 8036, Graz, Austria; 2 Department of Psychiatry, Ludwig-Maximilians University of Munich, Nußbaumstraße 7, 80336, Munich, Germany

**Keywords:** Solid-organ transplantation, Overall mental distress, Health-related quality of life

## Abstract

**Background:**

Our retrospective follow-up study aimed to explore the degree of overall mental distress in a cohort of solid-organ transplantation (SOT) recipients after liver, heart or lung transplantation. Furthermore, we investigated how overall mental distress is linked to health-related quality of life.

**Methods:**

123 SOT patients treated during the study period were enrolled in this investigation at a mean of 24.6 months (SD=11.6) after transplantation. Before transplantation, the Transplant Evaluation Rating Scale (TERS) was used to classify the level of adjustment in psychosocial functioning among transplantation candidates. After transplantation, recipients completed a research battery, which included the SCL-90-R, and the SF-36.

**Results:**

39 (31.7%) transplantation recipients had clinically significant overall mental distress as measured on the Global Severity Index of the SCL-90-R. Obsessive-compulsive symptoms (92.3%), somatization symptoms (87.2%), anxiety symptoms (84.6%), depression symptoms (82.1%) and phobic anxiety symptoms (69.2%) were a frequent finding.

Transplantation recipients with overall mental distress had significant lower levels of adjustment in psychosocial functioning before transaplantation than those without overall mental distress as measured in the TERS. Transplantation-related overall mental distress symptomatology was associated with maximal decrements in health-related quality of life.

**Conclusion:**

Transplantation recipients may face major transplantation- and treatment-related overall mental distress and impairments to their health-related quality of life. Further, overall mental distress is a high-risk factor in intensifying impairments to patients’ overall quality of life.

## Background

Great achievements have been made in the field of transplantation surgery over the past decades [1]. However, these usually life-saving interventions also present those affected with stressful experiences and great demands that not infrequently also trigger consequent psychiatric illnesses. Thus affective illnesses, maladjustment and severe anxiety have been diagnosed in 19-54% of patients during psychiatric evaluation processes [2-11].

In the assessment of outcomes following solid-organ transplantation, the transplantation’s influence on psychic health and well-being is increasingly moving into the foreground [1,7,12-17]. A number of outcome studies showed that solid-organ transplantation is associated with improvements in health-related quality of life relative to the pretransplant period, but without restoring the health status levels described in the general population [10]. Earlier investigations showed a significant connection between psychiatric morbidity and impaired health-related quality of life [3].

The transplantation itself and the intensive care unit stay might be traumatic stressors that decrease health-related quality of life and can trigger overall mental distress. The term overall mental distress is widely used by mental health practitioners, to summarize a range of symptoms and experiences of a person's internal life that are commonly held to be confusing, troubling, or out of the ordinary. Overall mental distress has a wider scope than the related term mental illness. Thus mental illness refers to a specific set of medically defined conditions. In contrast a person in overall mental distress may display psychiatric symptoms such as depression, anxiety, confused emotions, hallucination or rage, without necessarily actually being ill in a psychiatric sense [18-20].

### Aims of the study

Our retrospective follow-up study aimed to explore the degree of overall mental distress in a cohort of solid-organ transplantation recipients after liver, heart or lung transplantation. Furthermore, we investigated how overall mental distress is linked to health-related quality of life. The well-validated Transplant Evaluation Rating Scale (TERS) [21] is well established to classify the level of adjustment in psychosocial functioning among transplantation candidates. However, it is not proof of wheter pretransplant evaluation based on the Transplant Evaluation Rating Scale can help in indicating patients who are at high risk of posttransplant overall mental distress and impairments in health-related quality of life. This research question is another research aim of this study. Furthermore, factors that are described in the literature as potential contributing or associated factors to overall mental distress and impairments in health-related quality of life such as substance abuse (benzodiazepines, alcohol abuse), postoperative medical complications, social support or medical complications are considered [10].

## Method

### Subjects and procedure

During the study period a total of 280 patients underwent transplantation at the Transplantation Center of the Klinikum Grosshadern, Ludwig-Maximilians-University, Munich, Germany.

Before transplantation, the psychometric observer-rating scale Transplant Evaluation Rating Scale (TERS) [21] was used to classify the level of adjustment in psychosocial functioning among solid-organ transplantation candidates. 65 of these 280 patients died before our retrospective follow-up study started. 215 eligible transplantation recipients were contacted by experienced psychiatrists and were asked to complete a research battery, which included an author-compiled clinical questionnaire, the SCL-90-R [22,23] and the Medical Outcome Study Form (SF-36) [24]. 123 transplantation recipients returned the questionnaires, at a mean of 24.9 months (SD=11.9) after transplantation. The SF-36 data were compared with data from normative population whose individuals were drawn at random with respect to age and gender from a large data base (n=3000) used for the validation of the SF-36 in Germany.

Collected transplantation characteristics were the indications for heart transplantation (ischemic or dilated cardiomyopathy), orthotopic liver transplantation (alcoholic liver disease, infectious hepatitis, primary biliary cirrhosis, malignancy or other), and lung transplantation (emphysema and chronic obstructive pulmonary disease, pulmonary fibrosis, pulmonary hypertension or other). Postoperative medical complications, acute rejections and a history of retransplantation were observed.

### Psychometric tests

Before solid-organ transplantation the transplantation candidates were evaluated by experienced consultation-liaison psychiatrists using the psychometric observer-rating TERS [21].

The TERS scale classifies the level of adjustment in psychosocial functioning among transplantation candidates and covers the different dimensions of psychosocial functions (pre-existing psychiatric morbidity, substance abuse, compliance, coping strategies, cognitive performance). The total sum reflects the current level of psychosocial functioning of the particular transplantation candidate. The higher the score, the worse the current level of psychosocial functioning.

After transplantation, an author-compiled questionnaire was used to obtain information about the patient and transplantation characteristics. Demographic variables included age, gender, years of education and/or vocational training, employment and marital status at the time of psychiatric assessment. The patient’s employment status was categorized as paid work (full- or part-time) or no paid work (disability, retired, unemployed). Marital status was categorized as married, single, divorced, or widowed. Further, preoperative chronic benzodiazepine consumption and preoperative alcohol abuse were recorded.

The Symptom Check List (SCL-90-R) [22,23] is a well-researched and frequently used multidimensional self-rating 90-item scale to screen for a broad range of psychological problems. Each of the 90 items is rated on a five-point Likert scale ranging from “not at all” to “extremely” relating to distress. The nine primary symptom dimensions are: Somatization, Obsessive-Compulsive, Interpersonal Sensitivity, Depression, Anxiety, Anger-Hostility, Phobic Anxiety, Paranoid Ideation and Psychoticism. The Global Severity Index is a global index that measures overall mental distress and the cut-off T-value for clinically significant overall mental distress is 60 [22].

To assess health-related quality of life, we applied the psychometrically well-validated German translation of the Medical Outcome Study Form (SF-36) [24], a 36-item self-rating questionnaire that covers eight health-related domains. The domains are Physical Functioning, Pain, General Health, Vitality, Social Functioning, Mental Health, Role Physical, and, Role Emotional. The Role Physical is defined as the extent to which the physical condition impairs work or other everyday activities (e.g. achieving less than usual, limitation of types of activities, or difficulty in carrying out certain activities). The Role Emotional is defined as the extent to which emotional problems impair work or other everyday activities.

Each domain yields a score ranging from 0 to 100 (best). In the vast majority of the published studies, the internal-consistency data of the SF-36 exceed 0.8 [24].

### Statistical analyses

Descriptive statistics were produced based on demographic, treatment-related and psychometric data (SCL-90-R, SF-36) and are presented as mean and standard deviation (SD) and median and range. The psychometric data of the SF-36 scores were not normally distributed as shown by the Kolmogorov-Smirnov Test. We therefore applied the non-parametric Mann–Whitney-U test to test differences between patients with or without a high degree of overall mental distress and the non-parametric Wilcoxon Test to compare transplantation recipients without a high degree of overall mental distress with healthy controls matched by age and gender. All statistic tests were two-tailed, with significance set at p<0.05. In case of multiple comparisons, an alpha adjustment (Bonferroni) was used and p values <0.006 were considered significant.

All statistical analyses were performed with SPSS 18.0 for Windows (SPSS; Chicago, IL).

The study was approved by the Institutional Review Board of the Ludwig-Maximilians-University of Munich. Data protection met the standards set by German law. All persons gave their informed consent prior to their inclusion in the study.

## Results

### Sociodemographic characteristics

All 123 participants (69.1% men, 30.9% women) were Caucasian, and the mean age was 52.6 (SD=11.6; Median: 57.0) years.

Respondents with complete data were similar to those lost for refusal and untracebility on the sociodemographic and treatment parameters.

Table 1 summarizes the pre-transplantation clinical characteristics of the whole sample.

**Table 1:** Indication for transplant

**Heart**	**Liver**	**Lung/heart and lung**
**(n = 60)**	**(n = 42)**	**(n = 21)**
Ischemic cardiomyopathy:	Alcoholic liver disease:	Emphysema/COPD:
34 (56.7%)	12 (28.6%)	6 (28.6%)
Dilatative cardiomyopathy:	Infectious hepatitis ^a^:	Pulmonary fibrosis:
26 (43.3%)	13 (31%)	7 (33.3%)
	Primary biliary cirrhosis:	Pulmonary hypertension:
	5 (11.9%)	3 (14.3%)
	Malignancy^b^:	Other^d^:
	6 (14.3%)	5 (23.8%)
	Miscellaneous^c^:	
	6 (14.3%)	

^a^ Infectious hepatitis encompasses HBV-related and/or HCV-related liver diseases.

^b^ Malignancy includes hepatocellular carcinoma and other hepatic malignancies.

^c^ Miscellaneous comprises Wilson’s disease, Budd-Chiari syndrome, fulminant hepatic failure, and cryptogenic cirrhosis.

^d^ Other indications for lung transplant include lymphangioleiomyomatosis, cystic fibrosis, and congenital cystic adenomatoid malformation of the lung.

Table 2 shows the post-transplantation sociodemographic characteristics of patients.

**Table 2:** Posttransplantation sociodemographic characteristics

**Category**	**All patients**	**Heart**	**Liver**	**Lung/heart and lung**	**p**^**d**^
	**(n = 123)**	**(n = 60)**	**(n = 42)**	**(n = 21)**	
***Gender***					
Male	85 (69.1%)	50 (83.3%9	28 (66.7%)	7 (33.3%)	χ^2^=18.393; df=2;
Female	38 (30.9%)	10 (16.7%)	14 (33.3%)	14 (66.7%)	**p<0.001**^**a**^
***Age***					
Mean (years)	52.6	54.9	50.9	49.5	
SD	± 11.6	± 11.2	± 11.8	± 11.2	p=0.038^b^
Median	57.0	58.0	54.5	53.0	
***Marital status***					
Single	12 (9.8%)	5 (8.3%)	2 (4.8%)	5 (23.8%)	p=0.151^c^
Married	93 (75.6%)	49 (81.7%)	31 (73.8%)	13 (61.9%)	
Widowed	2 (1.6%)	1 (1.7%)	1 (2.4%)	-	
Divorced	16 (13%)	5 (8.3%)	8 (19%)	3 (14.3%)	
***Employment status***					
Full-time	25 (20.3%)	11 (18.3%)	13 (31.0%)	1 (4.8%)	**p=0.006**^**c**^
Part-time/homeworker	12 (9.8%)	4 (6.7%)	8 (19%)	-	
Unemployment	4 (3.3%)	3 (5%)	1 (2.4%)	-	
Retired	12 (9.8%)	9 (15%)	2 (4.8%)	1 (4.8%)	
Disabled from work due to health-related reasons	70 (56.9%)	33 (55%)	18 (42.9%)	19 (90.5%)	

*SD*=Standard deviation.

^a^ χ^2^ tests.

^b^ Kruskal-Wallis one-way analysis of variance or ranks.

^C^ Fisher’s exact test.

^d^Statistical tests were performed to proof differences of probability or categorical distributions among the different kinds of transplantation (liver-, heart-, lungtransplantation).

### Psychiatric morbidity before transplantation

Before transplantation the psychometric observer-rating scale TERS was performed on all transplantation candidates, and the following psychiatric diagnoses were recorded: alcoholism, abstinent at the time of psychiatric assessment (2.4%), alcohol abuse, abstinent at the time of psychiatric assessment (7.4%), adjustment disorder (17.2%), substance abuse (opiate, benzodiazepine) (4.9%), dysthymia (0.8%) and obsessive-compulsive disorder (0.8%). These diagnoses have been based on a psychiatric clinical interview.

### Overall mental distress after solid-organ transplantation

After transplantation 39 (31.7%) recipients had clinically significant overall mental distress as measured on the Global Severity Index of the SCL-90-R (SCL-90-R cut-off T-value for clinically significant overall mental distress: 60). The mean Global Severity Index score for the transplantation recipients with clinically significant overall mental distress was 70.6 (SD=7.3; Median: 70.0) and for those without clinically significant overall mental distress 46.3 (SD=7.4; Median: 47.5) [Mann–Whitney-U=0.000; p<0.001].

Reflecting the primary symptom dimensions of the SCL-90-R, transplantation recipients with clinically significant overall mental distress mainly had significant values in the following dimensions: Obsessive-compulsive (92.3%), Somatization (87.2%), Anxiety (84.6%), Depression (82.1%) and Phobic Anxiety (69.2%). Less frequent findings were high values in these dimensions: Psychoticism (61.5%), Paranoid Ideation (59%), Anger and Hostility (53.8%) and Interpersonal Sensitivity (51.3%).

No significant differences between transplantation recipients with clinically significant overall mental distress and those without were found in the following sociodemographic characteristics: age (Mann–Whitney-U=1541.0; p=0.598), gender (χ^2^ =0.670; df=1, p=0.413), maritial status (χ^2^ =1.7; df=3, p=0.636), years in education and/or vocational training (Mann–Whitney-U=1562.5; p=0.675). Regarding employment status, transplantation recipients with clinically significant overall mental distress were less often employed than those without such symptomatology (χ^2^ =9.9; df=1, p=0.04).

No significant differences between patients with postoperative overall mental distress symptomatology and those without were found in the pre- and postoperative frequency of alcohol abuse (Fisher’s exact test; p=0.585).

Table 3 illustrates the pre- and postoperative frequency of alcohol abuse.

**Table 3:** Pre- and postoperative frequency of alcohol abuse

	**No alcohol abuse before and after SOT**		**Alcohol abuse before SOT**		**Current alcohol abuse after SOT**		**p**
	**n**	**%**	**n**	**%**	**n**	**%**	
**SOT recipients with high degree of overall mental distress**	28	71.8%	9	23.1%	2	5.1%	p=0.585^a^
**SOT recipients with low degree of overall mental distress**	67	79.8%	14	16.7%	3	3.6%	

SOT: solid-organ transplantation.

^a^ Fisher’s exact test.

Transplantation recipients with overall mental distress more frequently take benzodiazepines after the surgical intervention. However, it needs to be mentioned that even before transplantation patients with postoperative overall mental distress symptomatology displayed a higher level of benzodiazepine consumption (Fisher’s exact test; p<0.001).

Table 4 shows the benzodiazepine consumption before and after transplantation.

**Table 4:** Benzodiazepine consumption before and after transplantation

	**No benzo-diazepine consumption before and after SOT**		**Benzodiazepine consumption before SOT**		**Present benzo-diazepine consumption after SOT**		**p**
	**n**	**%**	**n**	**%**	**n**	**%**	
**SOT recipients with high degree of overall mental distress**	24	61.5%	5	12.8%	10	25.6%	p<0.001^a^
**SOT recipients with low degree of overall mental distress**	77	91.7%	1	1.2%	6	7.1%	

*SOT*: solid-organ transplantation.

^a^ Fisher’s exact test.

### Overall mental distress and postoperative medical complications

In the sample we studied, the occurrence of postoperative medical complications (e.g. bleeding, infections, cardio-vascular, rejection) did not significantly differ in transplantation patients with postoperative overall mental distress from those without overall mental distress (χ^2^ =3.202; df=1, p=0.074). Transplantation recipients with postoperative overall mental distress did not show acute rejections following transplantation more frequently than those without overall mental distress (Fisher’s exact test; p=0.330).

Table 5 presents the history of retransplantations.

**Table 5:** The history of retransplantations of SOT recipients

	**No retransplantation**		**One retransplantation**		**Two retransplantations**		**p**
	**n**	**%**	**n**	**%**	**n**	**%**	
**SOT recipients with high degree of overall mental distress**	37	94.9%	1	2.6%	1	2.6%	p=0.330^a^
**SOT recipients with low degree of overall mental distress**	79	94.0%	5	6%	0	0%	

*SOT*: solid-organ transplantation.

^a^ Fisher’s exact test.

### Overall mental distress and the type of transplantation

There was no significant difference in the frequency of overall mental distress after transplantation between patients with liver, heart, or lung transplantation (χ^2^ =1.411; df=2; p=0.534).

Table 6 illustrates the overall mental distress symptomatology according to type of transplantation.

**Table 6:** Overall mental distress according to type of transplantation

	**Liver transplantation**		**Heart transplantation**		**Lung transplantation**		**p**
	**n**	**%**	**n**	**%**	**n**	**%**	
**SOT recipients with high degree of overall mental distress**	15	35.7%	16	26.7%	8	38.1%	χ^2^ =1.411; df=2; p=0.534^a^
**SOT recipients with low degree of overall mental distress**	27	64.3%	44	73.3%	13	61.9%	

SOT: solid-organ transplantation.

^a^ χ^2^ test.

### Overall mental distress and posttransplant type of medication

Patients with overall mental distress after transplantation did not differ at the time of exploration in their prescribed medication from those recipients without postransplant overall mental distress (tacrolimus, cyclosporine [χ^2^ =3.362; df=4; p=0.499]; steroids [χ^2^ =1.526; df=1; p=0.217]).

### The level of adjustment in psychosocial functioning before transplantation and overall mental distress after transplantation

Transplantation recipients with clinically significant overall mental distress had significantly lower levels of adjustment in psychosocial functioning before transplantation than those without significant overall mental distress after transplantation as measured in the TERS (Mann–Whitney-U=1255; p=0.033). The mean TERS score before transplantation was 36.4 (SD=9.8) in the subgroup of transplantation recipients with clinically significant overall mental distress and 32.8 (SD=7.5) in transplantation recipients without significant overall mental distress.

No differences between patients with clinically significant postoperative overall mental distress symptomatology and those without were found in the preoperative frequency of psychiatric disorders (Fisher’s exact test; p=0.237).

Table 7 Preoperative frequency of psychiatric disorders.

**Table 7:** Preoperative frequency of psychiatric disorders

	**No preoperative psychiatric disorder**		**Preoperative psychiatric disorder**		**p**
	**n**	**%**	**n**	**%**	
**SOT recipients with high degree of overall mental distress**	32	82.1%	7	17.9%	p=0.237^a^
**SOT recipients with low degree of overall mental distress**	76	90.5%	8	9.5%	

SOT: solid-organ transplantation.

^a^ Fisher’s exact test.

### The level of adjustment in psychosocial functioning before transplantation and postoperative medical complications and acute rejection

Transplantation recipients with postoperative medical complication did not differ in the level of adjustment in psychosocial functioning before transplantation as measured by the TERS (Mann–Whitney-U: 1614.5, p=0.160). However, transplant recipients with acute rejection had significantly lower TERS scores before transplantation, than those without rejection, indicating a lower level of adjustment in psychosocial functioning before transplantation (pretransplant TERS mean score in the subgroup of recipients with rejection: 33.28, pretransplant TERS mean score in the subgroup of recipients without rejection: 36.68; Mann–Whitney-U: 593.5, p=0.027).

### Health-related quality of life and overall mental distress after transplantation

In comparison with those transplantation recipients without clinically significant overall mental distress, transplantation recipients suffering from overall mental distress displayed significant impairments in all health-related quality of life SF-36 domains: Physical Functioning, Role Physical, Pain, General Health, Vitality, Social Functioning, Role Emotional and Mental Health.

Table 8 shows the health-related quality of life SF-36 domains according to overall mental distress.

**Table 8:** HRQOL SF-36 subtests for SOT recipients with high degree of overall mental distress and SOT recipients with low degree of overall mental distress

	**SOT recipients with high degree of overall mental distress**	**SOT recipients with low degree of overall mental distress**	**Mann–Whitney-U**	**p**
Physical Functioning	Mean: 51.3	Mean: 70.5	894.00	**p<0.001**^**a**^
	SD: ± 27.6	SD: ± 25.9)		
	Median: 57.5	Median: 80.0		
	Minimum: 0	Minimum: 0		
	Maximum: 95	Maximum: 100		
Role Physical	Mean: 31.6	Mean: 63.1	927.50	**p<0.001**^**a**^
	SD: ± 37.1	SD: ± 43.4		
	Median: 12.5	Median: 100.0		
	Minimum: 0	Minimum: 0		
	Maximum: 100	Maximum: 100		
Pain	Mean: 43.2	Mean: 73.7	701.00	**p<0.001**^**a**^
	SD: ± 26.3	SD: ± 28.3		
	Median: 41.0	Median: 74.0		
	Minimum: 0	Minimum: 0		
	Maximum: 100	Maximum: 100		
General Health	Mean: 42.2	Mean: 63.5	652.00	**p<0.001**^**a**^
	SD: ± 17.8	SD: ± 20.3		
	Median: 41	Median: 62.0		
	Minimum: 0	Minimum: 0		
	Maximum: 82	Maximum. 100		
Vitality	Mean: 40.7	Mean: 60.3	719.00	**p<0.001**^**a**^
	SD: ± 17.8	SD: ± 20.09		
	Median: 45.0	Median: 60.0		
	Minimum: 5	Minimum: 0		
	Maximum: 80	Maximum: 100		
Social Functioning	Mean:65.8	Mean: 86.7	701.00	**p<0.001**^**a**^
	SD: ±20.9	SD: ± 19.7		
	Median: 62.5	Median: 100.0		
	Minimum: 25	Minimum: 0		
	Maximum: 100	Maximum: 100		
Role Emotional	Mean: 53.5	Mean: 85.8	930.00	**p<0.001**^**a**^
	SD: ± 44.2	SD: ± 31.9)		
	Median: 50.0	Median: 100.0		
	Minimum: 0	Minimum: 0		
	Maximum: 100	Maximum: 100		
Mental Health	Mean: 59.9	Mean: 79.8	578.00	**p<0.001**^**a**^
	SD: ± 19.0	SD: ± 16.3)		
	Median: 60.0	Median: 84.0		
	Minimum: 12	Minimum: 0		
	Maximum: 96	Maximum: 100		

SOT: solid-organ transplantation.

^a^ Mann–Whitney-U-test.

Compared with healthy controls matched by age and gender, transplantation recipients without significant overall mental distress showed impairments in some health-related quality of life SF-36 domains: Physical Functioning (Wilcoxon-Z=−6.239, p<0.001), Role Physical

(Wilcoxon-Z=−5.115, p<0.001), Pain (Wilcoxon-Z=−3.780, p<0.001), General Health (Wilcoxon-Z=−3.009, p<0.001), Vitality (Wilcoxon-Z=−3.060, p=0.002) and Social Functioning (Wilcoxon-Z = −3.338, p=0.001).

### The level of adjustment in psychosocial functioning before transplantation and health-related quality of life after transplantation

A Spearman rank correlations between pretansplant TERS scores and postoperative quality of life SF-36 domains scores showed that a low level of adjustment in psychosocial functioning before transplantation (indicated by higher pretansplant TERS scores) is significant negatively correlated with posttransplant health-related quality of life in the following SF-36 domains: Vitality (r_s_=−0.239, p=0.009), Role Emotional (r_s_=−0.239, p=0.009), Social Functioning (r_s_=−0.182, p=0.046), and Mental Health (r_s_=−0.325, p<0.0001). In addition a trend of a negative correlation between high pretansplant TERS scores and pottransplant low health-related quality of life was observed in the SF-36 domains: Role emotional (r_s_=−0.176, p=0.055) and Pain (r_s_=−0.157, p=0.086). No significant correlations between pretansplant TERS scores and pottransplant health-related quality of life were detected in the SF-36 domains Physical Functioning (r_s_=−0.069, p=0.453) and General Health (r_s_=−0.131, p=0.153).

## Discussion

### Overall mental distress

In spite of great advances in transplantation surgery and constant improvement in surgical techniques, tissue matching, organ preservation and patient selection, many patients display symptoms of mental distress and psychiatric morbidity [1-4,6,8-10]. However, up to now there have only been a few empirical findings on the prevalence of overall mental distress in transplantation recipients [25-27].

In our study, a large number (n=39 [31.7%]) of transplantation recipients displayed clinically significant overall mental distress independent of prescribed posttransplant medication (tacrolimus, cyclosporine, steroids), and in the SCL-90-R obsessive-compulsive symptoms (92.3%), somatization symptoms (87.2%), anxiety symptoms (84.6%), depression symptoms (82.1%) and phobic anxiety symptoms (69.2%) were a frequent finding.

Obsessive-compulsive symptoms could be regarded as thoughts that are repeated reminders of the transplantation and the subsequent stay in the intensive care unit. Concentration difficulties and an impaired ability to work are also shown in this SCL-90-R dimension. Furthermore, it could be hypothesized that the postransplant necessity for being very accurate in taking the prescribed medication to avoid rejections may aggravate obsessive-compulsive symptoms. Intrusive memories are also frequently mentioned in other studies as post-traumatic symptoms after transplantation [28]. For example, in an orthotopic liver transplantation study by Rothenhäusler et al. [10], 2.7% suffered from full post-traumatic stress disorder and 16% from partial posttraumatic stress disorder. In another study by Favaro et al. [29], the estimated frequency of transplantation-related posttraumatic stress disorder after heart transplantation was 12%.

Somatization symptoms could be interpreted on the one hand as greater body awareness after transplantation, while on the other hand they could reflect increased self-monitoring of the body due to traumatic illness and transplantation experiences.

Depressive and anxiety symptomatologies are a frequent finding after transplantation and have been reported in numerous studies [4,6,8-10,13,30,31].

Deficits in interpersonal sensitivity (51.3%) as measured in the SCL-90-R subscale interpersonal sensitivity are a less frequent finding in our study’s patients and seem to show the physical vulnerability and insecurity remaining after the life-threatening experiences of illness and transplantation. The physical impairments and resulting feelings of low self-confidence could therefore cause of withdrawal from social life. In the case of transplantation recipients the psychoticism subscale of the SCL-90-R describes rather the isolation and the feeling of depersonalization after transplantation and should not be primarily interpreted as measurement of psychotic symptoms. Such symptoms are reported by 61.5%. Paranoid ideation (59%) in the SCL-90-R reflect the sense of inferiority and mistrust after transplantation, and the primary symptom dimension Anger and Hostility (53.8%) characterizes the irritability and unbalance in many transplantation recipients.

Regarding our results it needs to be mentioned that transplantation candidates with low levels of adjustment in psychosocial functioning before transplantation are at higher risk of developing overall mental distress symptomatology after transplantation, and the incidence of overall mental distress is independent of type of transplantation and postoperative medical complications. However, transplant recipients with acute rejection have significantly lower TERS scores before transplantation than those without rejection, indicating a lower level of adjustment in psychosocial functioning before transplantation. Furthermore, no significant differences between patients with postoperative overall mental distress symptomatology and those without were found in the pre- and postoperative frequency of alcohol abuse. However, transplantation recipients with overall mental distress displayed a higher level of benzodiazepine consumption before and after the surgical intervention.

### Overall mental distress and health-related quality of life

Health-related quality of life is an important measure of outcome after transplantation and in recent years, several studies have demonstrated that transplantation has beneficial effects on quality of life for the majority of patients, both in intermediate and in longterm outcomes [10,32,33]. However, impairments in health-related quality of life have been diagnosed in transplantation recipients with some psychiatric disorders (e.g. anxiety and affective disorders, posttraumatic stress disorder) after transplantation [25-28,33-35]. In this present study even transplantation recipients without overall mental distress display more impairments in some health-related quality of life domains than healthy controls matched by age and gender. It is recognised that overall mental distress impairs health-related quality of life and life satisfaction even in people with overall good health. Therefore a major aim of the present study was to explore if overall mental distress in solid-organ transplantation recipients is strongly associated with massive additional impairments to health-related quality of life. Indeed the transplantation recipients with overall mental distress examined in this study had a lower health-related quality of life compared with transplantation recipients without overall mental distress as shown by impairments in all health-related quality of life SF-36 domains.

Furthermore, a low level of adjustment in psychosocial functioning before transplantation (indicated by higher pretansplant TERS scores) was significantly negatively correlated with posttransplant health-related quality of life in the SF-36 domains Vitality, Role Emotional, Social Functioning, and Mental Health. In conclusion our results suggest that overall mental distress in solid-organ transplantation recipients is strongly associated with massive additional impairments to health-related quality of life. These results allow for the hypotheses that overall mental distress has adverse health effects that impair coping with activities in daily life, and/or that transplantation recipients with overall mental distress symptoms already display greater health impairments as such and not just after the transplantation.

### Limitations

There are several limitations to our study. First, the format of the study is a retrospective follow-up. Further, the small group sizes of the organ-subgroups may be a limitation that needs to be mentioned. Regarding the statistical analysis the different organ-subgroups have been considered as a whole group, in spite of their diverse characteristics (e.g. development of organ insufficiency, different waiting times, different availability of assisting devices). However, several studies have shown that a common data interpretation is possible due to the homogeneously traumatic experiences caused by the solid-organ transplantation followed by intensive care unit treatment [28]. All participating participants were Caucasian. As a limitation some ethnic groups may have different interpretations of the symptoms.

Finally, our observations should be confirmed in studies with higher sample sizes to gain a clearer insight into the degree of overall mental distress in transplantation recipients.

## Conclusions

Transplantation recipients may face major transplantation- and treatment-related overall mental distress and impairments to their health-related quality of life. Further, overall mental distress is a high-risk factor in intensifying impairments to patients’ overall quality of life.

The results of our study indicate that a pretransplant evaluation based on the Transplant Evaluation Rating Scale can help in indicating patients who are at high risk of posttransplant mental distress and impairments in health-related quality of life. Therefore pretransplant diagnostic screening based on the Transplant Evaluation Rating Scale may facilitate postransplant psychosocial support for these patients, who could be given specialized help in advance of transplantation. Putatively this would also have an impact on the transplant recipients’ postoperative health-related quality of life.

In conclusion we advise that as part of routine clinical care, an early and comprehensive diagnosis and bio- and psychosocial therapeutic treatment of transplantation patients with clinically significant concomitant overall mental distress to be carried out, so that their overall mental distress symptomatology can be treated rapidly and their quality of life can be improved. In the future the development of new specific therapeutic strategies to reduce overall mental distress are desirable.

## Abbreviations

SCL-90-R: SCL-90-R Symptom Check List; SD: Standard deviation; SF-36: Medical Outcome Study Form SF-36; TERS: Transplant Evaluation Rating Scale (TERS).

## Competing interests

The authors declare that they have no competing interests.

## Authors’ contributions

AB: participated in data analysis; participated in the writing of the paper. TK: participated in research design; participated in the performance of the research. H-BR: participated in research design; participated in the performance of the research, participated in the writing of the paper. All authors read and approved the final manuscript.

## Authors’ information

Department and Institution where work was performed:

Psychiatric Consultation-Liaison Service, Department of Psychiatry, Ludwig-Maximilians-University Medical School and Klinikum Grosshadern, Munich, Germany.
